# Implementation of the Milan System for Reporting Salivary Gland Cytology: A Two-Year Outcome Cytopathology Data of a Tertiary Care Center

**DOI:** 10.7759/cureus.60842

**Published:** 2024-05-22

**Authors:** Soudamini Mahapatra, Chinmaya Sundar Ray, Aparajita Mishra, Dileswari Pradhan

**Affiliations:** 1 Department of Hematology, Institute of Medical Sciences and SUM Hospital, Bhubaneswar, IND; 2 Department of ENT, Pandit Raghunath Murmu Medical College & Hospital, Baripada, IND; 3 Department of Pathology, Government Medical College and Hospital, Sundargarh, Sundargarh, IND; 4 Department of Pathology, Srirama Chandra Bhanja (SCB) Medical College, Cuttack, IND

**Keywords:** functional neuroimaging, risk stratification, salivary gland swelling, milan system, cytopathology

## Abstract

Objective: In 2015, the Milan System for Reporting Salivary Gland Cytopathology (MSRSGC) was implemented to eliminate overlapping and disparate morphologies in salivary gland lesions. This approach helps track diagnostic findings, describe the risk of malignancy for each group, and advance therapy based on the results. The research aimed to classify fine-needle aspiration (FNA) smears, analyze malignancy risk, correlate cytology with histological diagnosis, and reduce unnecessary surgeries.

Methodology: We evaluated 217 individuals using FNA, classified their conditions using the Milan System, and followed up on 149 cases through histopathology. Both the risk of malignancy in each cluster and the total risk of malignancy were noted.

Results: The most recent studies, as reported by the MSRSGC, found almost universal agreement about this grouping. The FNA cytopathology test demonstrated a sensitivity of 75% for identifying salivary gland abnormalities and a specificity of 93.16%. The findings indicated that the test had an accuracy of 89.66%, with a positive predictive value of 72.41% and a negative predictive value of 93.97%.

Conclusion: The MSRSGC offers a standardized technique for examining the results and assists the physician in determining the treatment plan that will be most beneficial.

## Introduction

The salivary gland neoplasm accounts for around 3-6% of all head and neck tumors and 3% of all malignancies [1,2]. Researchers calculated the incidence of malignant and benign tumors in the salivary gland to be 0.2-9.7 and 1.1-2.9 per 100,000 individuals, respectively [3,4]. Fine-needle aspiration (FNA) is a highly successful and minimally invasive diagnostic procedure widely recognized as the best and most well-established first-line option. Furthermore, it enjoys widespread acceptance among patients and leads to a reduced incidence of postoperative complications. It is susceptible and specific, with sensitivity values ranging from 54% to 98% and specificity values ranging from 88% to 98% [5,6]. FNA can help distinguish between non-neoplastic (NN) and malignant lesions. Distinguishing between benign and malignant tumors is valuable within the neoplastic classification. The FNA test is crucial for identifying malignant tumors since it can accurately determine low- and high-grade malignancies [5,7]. Different lesions require different therapeutic strategies, with NN lesions managed predictably and neoplastic lesions addressed by surgical intervention.

FNA is valuable for assessing the extent of necessary surgical intervention, such as determining whether neck dissection and facial nerves can be preserved [8-10]. Salivary gland cytology, despite its numerous benefits, is a challenging field in cytopathology. The main problems with salivary gland cytology were that there wasn't a way to measure the extent of invasion, the lesions weren't all the same, and there were noticeable similarities in their appearance [8,10]. As a result, in 2015, the American Society of Cytopathology and the International Academy of Cytology endorsed a six-stage classification scheme [10-13]. To meet the criteria for appropriate treatment recommendations and establish a uniform approach to reporting cytopathology findings in the salivary glands among collaborating institutions, they introduced a new reporting system, the Milan System for Reporting Salivary Gland Cytopathology (MSRSGC). Additionally, the creation of this system aimed to satisfy the criteria for providing practical recommendations for therapeutic interventions [12,13]. This study aimed to evaluate the effectiveness of the MSRSGC in tertiary care, categorizing samples of salivary gland lesions into groups and monitoring the instrument's performance.

## Materials and methods

From August 2017 to July 2019, the Departments of Pathology and Ear, Nose, and Throat (ENT) at SCB Medical College conducted a study with approval number SCB/1898/19. This study included a population of 217 individuals, out of which 149 patients received a histological follow-up. Each patient with salivary gland lesions, referred from the ENT outpatient department, had their radiological and early clinical histories recorded, and the study collected data on their age, gender, duration, onset, presence or absence of pain, and recurrence. Each patient underwent a meticulous clinical assessment of the lesion, documenting its location, dimensions, quantity, texture, attachment to the underlying structure, and any deviation from the normal condition of the mouth. We performed the aspiration using a 10-cc disproven syringe with a 22G needle, making one to four passes. The number of passes varied depending on the lesion's size and complexity. We followed strict aseptic protocols and obtained consent from the participating study groups before beginning. We palpated the lesion, immobilized it with two fingers, and then aspirated it. We created three smears using the aspirated materials, fixing two with alcohol and air-drying one. To stain the air-dried slides, we used the Diff Quik method, as well as the hematoxylin and eosin (H&E) and pap stain techniques to color the alcohol-fixed smears. The Milan System employed terminology to classify them into the six groups outlined in Table 1. 

**Table 1:** Categories of the Milan System, implied risk of malignancy, and recommended clinical management a: Specimen adequacy criterion has not been validated, b: subset of patients may be followed clinically, c: intraoperative consultation may be helpful to determine the extent of surgery, and d: extent of surgery depends on the type and grade of malignant tumor. FNA: Fine-needle aspiration

Serial no:	Diagnostic category	Risk of malignancy (ROM) (%)	Management
1	Non-diagnostic ^a^	25 [0-67%]	Clinical and radiological correlation/repeat FNA
2	Non-neoplastic	10 [0-20%]	Clinical follow-up and radiological correlation
3	Atypia of undermined significance (AUS)	20 [10-35%]	Repeat FNA and surgery
4	Neoplasm		
A	Benign	<5 [0-13%]	Surgery and clinical follow-up^b^
B	Salivary gland neoplasm of uncertain malignant potential (SUMP)	35 [0-100%]	Surgery^c^
5	Suspicious of malignancy (SM)	60 [0-100%]	Surgery ^c^
6	Malignant (M)	90 [35-100%]	Surgery ^c,d^

We used a comprehensive description of the approach to calculate the overall risk of malignancy (OROM) and the risk of malignancy (ROM) for each group. "Risk of malignancy" refers to the proportion of FNAs in a specific group with malignant outcomes compared to the total number of FNAs that underwent follow-up evaluations. Similarly, we found OROM by dividing the percentage of FNA that were later found to be malignant by the total number of FNA, regardless of whether they were tested later or not. However, the OROM represents the proportion of FNA ultimately determined to be malignant following additional examination. Surgery was performed on the follow-up instances, and a correlation between the histology and cytology was discovered during processing.

## Results

The patient group for this study included 217 people: 144 males and 73 females. The minor salivary glands were responsible for 52 of these occasions; the submandibular gland was responsible for 68 of these instances, and the parotid gland was responsible for 97. Each of these individuals had visible lesions. We performed a histological examination on 149 people and found that the majority, or 131 instances, were not malignant. Next, 52 cases make up the benign neoplastic category. The non-diagnostic (ND) group consisted of five instances (2.3%) that met the requirement of being less than 10%. We identified the ND group as having inadequate cellularity, no substantial cells in the aspirates, and patients unwilling to cooperate. We performed a histological follow-up on four instances, and except for one, all proved to be NN. We assigned one case to this group due to the absence of cellularity and excessive fluid on re-aspiration, both of which occurred in the presence of a clinically and radiologically diagnosed deep-seated lesion. We determined the patient's cancer to be inferior mucoepidermoid carcinoma after a second round of aspiration under ultrasound guidance. Most of the 149 histological follow-up cases fell into the NN group, followed by the benign neoplasm group, with a low cancer risk of 2.8% and 1.9%, respectively. Atypia of uncertain significance (AUS), suspicious of malignancy (SFM), and salivary gland neoplasm of uncertain malignant potential (SUMP) category were all in "indeterminate" group. These findings did not prove beyond a reasonable doubt that the salivary gland tumors were malignant or benign. These findings led us to concentrate on examining the groups for the ROM analysis. The NN category comprises 131 cases or 60.4% of the total. The classification revealed chronic sialadenitis as the most common lesion, accounting for 31.8% of all cases. Retention cysts (18.4%) and sialadenosis (6.9%) came next. Sixty-five had histological follow-ups; of these, 55 had NN changes, 4 had benign neoplastic changes, and 6 had malignant changes. Further examination revealed that six of the cases were cancerous. Mucoid aspiration and insufficient cellularity were the most common reasons for a false-negative result when diagnosing retention cysts and chronic sialadenitis, as identified in six out of eight patients. Subsequent examination revealed a low-grade mucoepidermoid carcinoma. One patient initially received a diagnosis of sialadenosis due to the absence of cellularity and the unremarkable appearance of the cells. However, further testing found that the patient had acinic cell cancer (Table 2).

**Table 2:** Categorization of the salivary gland lesions as per the Milan System

Category	Number of cases N (%)
Non-diagnostic	5 (2.3%)
Non-neoplastic	131 (60.4%)
Chronic sialadenitis	69 (52.6%)
Retention cyst	40 (30.5%)
Sialodnosis	15 (11.4%)
Reactive hyperplasia of intraparotid lymph node	3 (2.29%)
Granulomatous lesion	2 (1.5%)
Abscess	2 (1.5%)
Atypia of undetermined significance	5 (2.3%)
4A Neoplastic benign	52 (24%)
Pleomorphic adenoma	39 (75%)
Warthin tumor	8 (15.3%)
Basal cell adenoma	1 (1.9%)
Hemangioma	1 (1.9%)
Oncocytoma	1 (1.9%)
Schwannoma	1 (1.9%)
Sebaceous adenoma	1 (1.9%)
4B Salivary gland neoplasm of uncertain malignant potential	4 (1.8%)
Suspicious of malignancy	4 (1.8%)
Malignant	16 (7.4%)
Adenoid cystic Ca	5 (31.2%)
Mucoepidermoid Ca	4 (25%)
Acinic cell Ca	3 (18.7%)
AdenoCa NOS	2 (12.5%)
Pleomorphic Ex Ca	1 (6.25%)
Salivary duct carcinoma	1 (6.25%)

We found five cases (2.3%) of AUS that did not meet the cytomorphological criteria and needed to be diagnosed further. All five cases underwent histological evaluation, and the findings revealed that two were carcinoma types: adenoid cystic carcinoma and low-grade mucoepidermoid carcinoma. We observed that 24 percent of cases were benign neoplastic tumors. We determined that the other was malignant, while the first was NN. The lack of chondromyxoid material-containing cells in the lesion led to the incorrect diagnosis of an adenoid cystic carcinoma instead of a pleomorphic adenoma, which led to an incorrect diagnosis of the condition. Further investigation showed that the initial diagnosis was false. Four incidents, accounting for 1.8% of all cases, reported salivary gland neoplasms with uncertain malignancies. The histological examination of each instance revealed two forms of cancer: a low-grade mucoepidermoid carcinoma and a pleomorphic adenoma. The aspiration revealed that the mucin was a mixture of intermediate-looking cells and chondromyxoid stroma.

According to the data in Table 3, only four cases (1.8% of the total) fall into the category of suspicious of malignancy, these patients were very suspicious of cancer, but there were no additional tests or evidence to support their suspicions. Histopathological analysis correctly classified a single case, previously labeled adenoid cystic carcinoma, as a basal cell adenoma. Table 3 shows that 16 cases, or 7.4% of the total, fall into the malignant category. One of these turned out to be a pleomorphic adenoma with squamous metaplasia after additional testing. In one case, the FNA cytology scan revealed a chondromyxoid stroma. The modest atypia of the cell indicated the presence of a pleomorphic adenoma. The patient had a history of recurrence, and the tumor had grown during the preceding month. A pleomorphic ex-carcinoma was the diagnosis made after additional research. A solitary instance that was initially diagnosed as adenoid cystic carcinoma was accurately categorized as a basal cell adenoma by histopathological investigation.

**Table 3:** Histological follow-up of Milan System diagnostic categories ND: Non-diagnostic, NN: non-neoplastic, AUS: atypia of undetermined significance, SUMP: salivary gland neoplasm of uncertain malignant potential, SFM: suspicious of malignancy, M: Malignant; ROM: risk of malignancy, OROM: overall risk of malignancy

Category	No. of cases	No. of cases with histological follow-up	Benign: non-neoplastic	Benign: neoplastic	Malignant	ROM	OROM
ND	05	04	03	00	01	25%	20%
NN	131	65	55	04	06	2.8%	4.5%
AUS	05	05	02	01	02	40%	40%
4A) Benign	52	51	01	49	01	1.9%	1.9%
4B) SUMP	04	04	02	01	01	25%	25%
SFM	04	04	--	01	03	75%	75%
M	16	16	--	01	15	93.7%	93.7%
Total	217	149	61	59	29		

## Discussion

FNA is widely accepted and effective because of its efficiency, speed, and minimal patient discomfort [14-16]. The goal of releasing MSRSGC in 2015 was to make it easier to correctly identify and treat abrasions in the salivary glands [12,13,16]. Empirical evidence supports this technique, establishing a link between diagnostic classifications and cancer probability, along with scientific treatment alternatives [13,16]. Before developing the Milan categorization technique, researchers proposed various alternative categories to enhance the clinical outcome of salivary gland neoplasm. Miller et al., put salivary lesions into five groups based on their levels: myxoid-hyaline, basaloid, oncocytic, lymphoid, and squamoid [9,14,16]. Tessy et al. classified the lesions into four categories: inflammatory, benign, malignant, and miscellaneous [15]. Griffith and colleagues employed a classification system with four levels. It encompassed the subsequent subcategories: benign, indicative of malignancy, neoplasm of uncertain malignant potential (NUMP), and affirmative for malignancies [16]. Nevertheless, classifying these groupings was challenging due to their rarity and overlapping morphological characteristics, complicating the task. Consequently, a descriptive report can create confusion among experts [17].

Our investigation indicated that the average age and distribution of genders in salivary gland lesions were similar to those observed in previous studies [18-20]. We designed the Milan System to improve communication among clinical professionals and institutions, thereby improving the quality of patient treatment [12,13,21]. The risk of malignancy for our category was 40% higher than projected. Rohilla et al. achieved a 100% risk of malignancy, while Rossi et al. observed a risk of malignancy in 43% of cases [22,23]. Individuals in this category should have radiological monitoring, re-aspiration, surgery, and histological follow-up to improve patient care. Upon examination, the mucin appeared to consist of chondromyxoid stroma and cells displaying intermediate characteristics. We can compare the current study's relative operating margin of 25% with the findings of Griffith et al. [24]. The "indeterminate" group consists of three conditions: SFM, SUMP, and AUS. We created this category as a classification for situations that were unclear and challenging to diagnose. The Milan System for reporting salivary gland cytopathology is a valuable tool for identifying these lesions and determining the most effective treatment approach [25,26].

Over two years, we systematically investigated the application of the Milan System for reporting salivary gland cytology on various types of salivary gland FNA specimens. Afterward, we carefully watched and assessed the outcomes compared to earlier studies [27]. Following the guidelines of the MSRSGC, we divided FNAs of the salivary gland into six groups. For each group, we calculated the ROM. The percentages, derived from the results of a previous investigation and the MSRSGC strategy, are as follows: 25%, 2.8%, 40%, 1.9%, 25%, 75%, and 93.7%. These findings were as accurate as, or potentially even more accurate than, the multiple reports received using traditional methods for diagnosing abnormalities in the salivary glands [28].

Limitations of the study

Our study has certain limitations, such as a small sample size, a shorter length for histological follow-up, and a retrospective nature, which undermine the credibility of our results. Conducting this research at a tertiary care center allows the analysis to potentially involve individuals with various ailments, such as neoplastic and non-neoplastic diseases and conditions.

## Conclusions

MSRSGC is not only logical and practical but also offers a flexible reporting approach that enhances patient care by promoting communication between pathologists and clinicians. The salivary gland lesion reporting system, which consists of six tiers, is user-friendly and reliable, rendering it valuable for assessing risk and designing treatment strategies. Our research recommends using MRSCG for all clinically identified salivary gland lesions to enhance treatment, prognosis, and patient outcomes.
